# Hospital reorganization at a public university hospital to address the COVID-19 pandemic

**DOI:** 10.1590/0034-7167-2024-0281

**Published:** 2025-09-08

**Authors:** Cleonice Roseli Ribeiro, Maria do Carmo Fernandez Lourenço Haddad, Flavia Mendonça da Silva Oussaki, Natacha Bolorino, Cremilde Aparecida Trindade Radovanovic, Maynara Fernanda Carvalho Barreto, Patrícia Aroni Dadalt

**Affiliations:** I Universidade Estadual de Londrina. Londrina, Paraná, Brazil; II Universidade Estadual de Maringá. Maringá, Paraná, Brazil; III Universidade Estadual do Norte do Paraná. Bandeirantes, Paraná, Brazil

**Keywords:** Hospital Restructuring, Hospital Administration, Hospitals, University, Pandemics, COVID-19, Reestructuración Hospitalaria, Administración Hospitalaria, Hospitales Universitarios, Pandemias, COVID-19

## Abstract

**Objectives:**

to highlight the strategies adopted by hospital managers in reorganizing managerial and care processes at a public university hospital in response to the COVID-19 pandemic.

**Methods:**

this is a single-case, cross-sectional, descriptive study with a qualitative approach. Data collection was conducted through document analysis and interviews with managers between March and June 2022, with an average duration of 30 minutes. The data were analyzed based on the assumptions of Donabedian’s Triad.

**Results:**

nine female managers participated in the study, aged between 40 and 60 years, with more than five years of experience in their positions. The thematic axes were grouped into three dimensions: structure, process, and outcome.

**Final Considerations:**

the work process was redefined according to the pandemic context, demonstrating the applicability of Donabedian’s Triad throughout the entire process of care and organizational restructuring at the studied institution.

## RESUMEN

**Objetivos:**

evidenciar las estrategias adoptadas por los gestores hospitalarios en la reorganización de los procesos de gestión y asistencia de un hospital universitario público para enfrentar la pandemia de COVID-19.

**Métodos:**

estudio de caso único, transversal y descriptivo, con un enfoque cualitativo. La recopilación de datos se realizo mediante análisis documental y entrevistas con gestores, llevadas a cabo entre marzo y junio de 2022, con una duración media de 30 minutos. Los datos fueron analizados a la luz de los supuestos de la Tríada de Donabedian.

**Resultados:**

participaron en el estudio nueve gestoras, con edades entre 40 y 60 años y más de cinco años de experiencia en el cargo. Los ejes temáticos se agruparon en las siguientes dimensiones: estructura, proceso y resultado.

**Consideraciones Finales:**

el proceso de trabajo fue redefinido según el contexto pandémico experimentado, evidenciando la aplicabilidad de la Tríada de Donabedian a lo largo de todo el proceso de reestructuración asistencial y organizativa de la institución estudiada.

## INTRODUCTION

Considered one of the greatest challenges of the 21st century, the COVID-19 pandemic created a high demand for emergency care within the Brazilian Unified Health System (SUS in Portuguese), leading to an unprecedented public health crisis in Brazil. The disease spread rapidly, affecting people in more than 100 countries across all five continents, prompting the World Health Organization (WHO) to declare a pandemic on March 11, 2020^(1)^.

The response to the COVID-19 pandemic required a swift reorganization of hospital services to ensure both effective healthcare delivery and management while also providing support for severe cases of the disease. This involved increasing the number of hospital beds within existing healthcare structures, as well as expanding and establishing new contingency hospitals to address the preexisting issue of hospital overcrowding^(2)^.

In this context, the Public Health Emergency Operations Center of Londrina, Paraná, Brazil, designated the University Hospital of the *Universidade Federal de Londrina* (HU-UEL in Portuguese) as a “reference hospital” in March 2020 to provide exclusive care for patients classified as “moderate” and “severe” cases. These cases were referred by the Mobile Emergency Care Service, the Integrated Emergency Trauma Care Service, and the State Bed Regulation Center^(3)^.

Given the challenges posed by the pandemic, hospital administrators and healthcare teams encountered numerous obstacles, necessitating the development of effective policies and management strategies to ensure adequate conditions for patient care within hospital settings^(4)^. Consequently, a gap was identified in the literature regarding studies that describe the experiences of hospital managers, which could serve as a reference for managing future pandemics and public health emergencies.

Based on this context, the following research question was formulated: How did the managers of a public university hospital implement and experience the processes of reorganizing managerial and care services in response to the COVID-19 pandemic?

## OBJECTIVES

To highlight the strategies adopted by hospital managers in reorganizing managerial and care processes at a public university hospital to address the COVID-19 pandemic.

## METHODS

### Ethical Aspects

This study complied with Resolution No. 510/2016, which provides guidelines for research involving human subjects^(5)^, and was approved by the Research Ethics Committee for Human Subjects. Participants signed the Informed Consent Form (ICF), and researchers signed the Confidentiality and Secrecy Agreement.

### Theoretical-Methodological Framework

The study adopted Avedis Donabedian’s triad as its theoretical and methodological framework, which considers three dimensions of quality in healthcare: 1) Structure, 2) Processes, and 3) Outcomes^(6, 7)^.

The first component, “structure”, encompasses human, physical, and financial resources used in healthcare provision, as well as organizational arrangements and funding mechanisms for these resources. The “process” component refers to the activities that constitute healthcare delivery and involve interactions between healthcare professionals and the assisted population. Lastly, “outcomes” refer to changes in the population’s health status resulting from the care received^(6, 7)^.

### Study Type

This is a single-case study with a qualitative approach and document analysis, conducted following the guidelines of the Consolidated Criteria for Reporting Qualitative Research (COREQ)^(8)^.

### Study Setting

The study was conducted at HU-UEL, considered the largest supplementary body of the State University of Londrina. Established in August 1971, HU-UEL serves patients from approximately 250 municipalities in Paraná and over 100 cities in other states. It has 419 beds, all allocated to the Brazilian Unified Health System (SUS) and distributed across various medical specialties^(3)^.

For the interviews, each manager’s office was used, with an average duration of 30 minutes per interview. To maintain confidentiality, the door remained closed, and only the participant and the researcher, who was properly trained for this purpose, were present.

### Data Source

The study population consisted of hospital managers from HU-UEL. The sample was obtained using the snowball sampling technique (Snowball Technique)^(9)^. As each manager completed their interview, they were asked to refer another manager who had participated in the hospital’s reorganization process to establish it as a reference hospital for COVID-19 care.

### Data Collection and Organization

For the interviews, a demographic and professional characterization form was used, containing information such as the directorate of operation, gender, age group, type of professional affiliation, years of experience in the profession, and years of experience in hospital management. Additionally, a semi-structured interview guide was applied, featuring a guiding question: “Tell me about the reorganization of work processes to address the COVID-19 pandemic in this hospital”. Auxiliary questions were formulated to obtain further insights into the work process if the participant did not provide sufficient information in response to the initial question. The interviews were recorded using both a mobile phone and a portable recorder.

A full transcription of the interviews was conducted using the Microsoft Word® version 365 audio-to-text converter to generate the first version of the verbal transcription, with audio description verification by two independent researchers.

To ensure participant anonymity, a coding system was employed using the letter “P” for participant, followed by a sequential Arabic numeral according to the order of the interviews: P1, P2, P3, etc.

Document data collection occurred simultaneously via the hospital’s intranet, known as Rede HIT. Managerial documents were retrieved to support the understanding of the contingency plan and the internal and external managerial and care flows. The relevant documents were saved in PDF format on the institutional computer and later transferred to a flash drive.

### Data Analysis and Statistics

Demographic and professional data were analyzed using descriptive statistics (simple frequency) via Microsoft Excel®. The interviews were analyzed based on Bardin’s framework^(10)^, structured into three stages: pre-analysis, material exploration, and result processing, leading to the emergence of three elements in alignment with Donabedian’s theoretical model.

## RESULTS

Nine managers participated in this study, working in the following directorates: Superintendency (n = 01), Nursing (n = 04), Administrative (n = 02), and Clinical (n = 02). All were women, aged between 40 and 60 years, with permanent employment as public servants, over 10 years of professional experience, and more than five years in hospital management positions.

The analysis of their statements revealed their experiences in addressing the COVID-19 pandemic, which were categorized into thematic axes and classified according to the structure, process, and outcome dimensions of Donabedian’s Triad^(6)^.

In light of Donabedian’s framework, within the structure dimension, the interviewees identified five thematic axes, as illustrated in the following statements.

### Moment when HU-UEL became a reference hospital for COVID-19 patient care

The managers recalled the historical moment when they were designated as a reference hospital for COVID-19 patient care, following the first reports from Wuhan, China:

*At the end of 2019, we were designated by the State Health Department as a reference for COVID-19 care in the northern region of the state and within the state’s healthcare network, alongside the four university hospitals in Paraná.* (P1)

*From the moment HU-UEL was designated as a reference hospital for COVID-19 care, we had to quickly implement many adjustments in work processes.* (P4)

### Planning for pandemic response

In this axis, participants reported adapting protocols from previous pandemics and revising existing work processes.

*The medical leadership adapted the care protocol from the H1N1 pandemic and determined that patients should be treated according to this protocol.* (P5)

*The planning of actions involved reviewing work processes, designing new care flows, restructuring services, extensive training, significant engagement, responsibility, perseverance, and team trust so that we could solidify this process together within HU-UEL.* (P1)

### Reorganization of physical infrastructure

At the onset of the pandemic, even though HU-UEL’s physical structure was considered adequate for a large tertiary public teaching hospital and a reference center for Londrina and the entire northern macro-region of Paraná, several infrastructure adjustments were necessary to establish it as a reference hospital for COVID-19 patient care.

*As soon as the hospital was designated as a reference hospital, funding was made available. The Emergency Department was expanded from 2,500 m^2^ to over 5,000 m^2^, and the construction of the new maternity ward was completed, which served as a contingency hospital during this period.* (P1)

Thus, the physical structure of HU-UEL, which previously comprised approximately 40,600 m^2^ of buildings, expanded to approximately 54,205.01 m^2^ in 2020, distributed across a 100,000 m^2^ site. In 2019, the institution’s installed capacity for hospitalizations across various medical specialties consisted of 294 beds. However, throughout the pandemic, by 2023, this number increased to 419 beds (a 29% increase), all allocated to SUS and distributed across care units.

In the expanded emergency department, there was a dedicated area for emergency care with beds designated for ambulatory hospitalization of patients with cardiovascular conditions. Due to the increased admission of COVID-19-positive patients, these beds had to be repurposed for COVID-19 care.

Additionally, the hospital annex originally intended for the new maternity ward, which was scheduled to open in 2020, was repurposed as a contingency hospital with a capacity of 100 beds to accommodate the increasing number of COVID-19 patients requiring hospitalization.

### Reorganization of human, administrative, and support resources

To meet the increased demand for services, it was necessary to hire additional professionals from both the healthcare sector and related support areas through public service contracts and to reallocate existing employees.

Before the pandemic, the institution had 355 statutory public servants and 261 contracted service providers, totaling 616 employees. To address the new demands, this number increased to 472 public servants and 777 outsourced workers, bringing the total workforce to 1,249 employees, representing a 101% increase in the active human resources contingent.

During the pandemic, the primary focus was on healthcare professionals. However, many administrative and support staff were also present in the hospital environment and played a crucial role in ensuring effective healthcare delivery, as highlighted by one participant:

*The administrative transport staff, who had never handled or transported a deceased patient before, stepped up to help. These were administrative drivers, and they had to deal with this situation.* (P7)

It is evident that both the increase in human resources and the expansion of administrative professionals’ responsibilities were key structural reorganization measures.

### Implementation of new services

The Community Health Assistance Division (DASC in Portuguese), a service established within the hospital in 2002 to provide healthcare to university staff and employees—offering medical, nursing, and dental care for elective or low-complexity cases—had to be expanded. This focus on worker well-being is reflected in the following statements:

*An institution cannot develop if it does not take care of its professionals. So, DASC received investments, and a psychiatrist and a cardiologist were hired to provide care for employees during the pandemic.* (P5)

*DASC took care of us and even our relatives, so we had the privilege of not having to wait in those long lines for medical attention.* (P5)

Within the process dimension, two thematic axes were identified, which are described below.

### Reorganization of Workflows and Processes

Several workflows were implemented, modifying work processes from the moment patients entered the emergency department for COVID-19 cases to their care and referral to internal sectors. These changes resulted from the efforts of a specialized team designated for this purpose, in addition to the implementation of cohort isolation for individuals with positive and negative diagnoses. Another strategy mentioned in the interviews was the adoption of a deliberative multiprofessional communication process (round), aimed at discussing best managerial and care practices.

*A working group was formed, composed of professionals from various areas, mostly department heads but also many specialists. That was where decisions were made. Many administrative meetings were held to define new patient care and examination workflows, establish entry criteria, and train human resources, as we were also uncertain about how the situation would unfold.* (P4)

*We tried to organize a workflow because we were initially disorganized. We designated specific locations to receive patients, identified suspected cases, and classified patients as low suspicion based on CT scans at the time. We also separated positive and negative patients and those with high suspicion, creating a well-structured workflow.* (P9)

*This reorganization was carried out through process reviews, workflow restructuring, extensive training, high engagement, responsibility, perseverance, and team trust, ensuring that we could consolidate this process together within HU-UEL.* (P1)

*Teamwork was what saved us. There was greater interaction among teams, and the round significantly improved communication between team members. I believe this was fundamental in ensuring we could provide care effectively.* (P9)

### Collection of information on the clinical characteristics of COVID-19

Patients affected by COVID-19 exhibited clinical characteristics with varying degrees of severity depending on their age group, as reported by participants. Additionally, it was revealed that disease progression to severe conditions fluctuated according to age, with unexpected clinical manifestations observed by the healthcare team.

*There were times when COVID-19 presented completely differently from what had been expected. It was severe in elderly patients and extremely critical in young patients. It was very difficult for everyone to witness young patients being intubated, many of whom did not survive. This was one of the most challenging aspects, and the residents often cried a lot.* (P9)

[...] C*OVID-19 patients exhibited what was considered ’silent hypoxemia,’ meaning their oxygen saturation was dropping while they were still awake. Sometimes, they were unaware of their shortness of breath, or we failed to fully grasp the severity of their condition and dyspnea.* (P9)

Within the results dimension, only one thematic axis emerged.

### Challenges, emotions, and lessons learned in managing the COVID-19 Pandemic

Weaknesses related to high-level management processes and team collaboration were mentioned, as one of the managers reported being at the beginning of their term when they had to face this challenge.

*It was a very turbulent administration in every sense. From the beginning, we faced several administrative setbacks, and then the pandemic came. We were immediately challenged to confront a serious problem.* (P2)

The emotions of other managers, healthcare professionals, and members of society were also highlightd.

*Gyms and bars were open while people were being hospitalized in critical condition—I started crying. My God, I can’t handle this.* [...]/have *nowhere else to place the bodies, the ventilators are running out, and people are at the bar. I panicked, I completely broke down.* (P7)

*Employees created a ‘crying spot’ in the Emergency Room, a place where staff would go to cry.* (P7)

*As the number of new COVID-19 cases declined, in addition to the legacy of infrastructure expansion and equipment acquisition, all the knowledge and experiences gained during this period remained.* (P9)

*If another pandemic arises, we will overcome it—we will do everything the same way.* (P2)

Based on these accounts, it became evident that overcoming COVID-19 was only possible due to the implementation of emergency strategies, which led to valuable learning experiences and the ability to overcome the challenges faced.

## DISCUSSION

The analysis of the reorganization process of managerial and care services at the institution under study was shaped by actions taken by senior management and the planning of new workflows.

Donabedian^(6)^ considers the structure dimension the most important, as it can influence the others, reinforcing the need for its monitoring, which includes all organizational aspects and material resources.

Notably, during the pandemic, the institution under study adjusted and reorganized its structures to accommodate patients with respiratory syndrome while also reevaluating the conventional in-person care model. This resulted in the implementation of several care strategies aimed at optimizing patient care and reducing the spread of infection without compromising necessary treatments.

In this context, the *Hospital das Clínicas at the Ribeirão Preto* Medical School (HCFMRP-USP) was also designated as one of the reference hospitals for COVID-19 treatment, requiring internal restructuring to meet the region’s demand. To achieve this, the hospital expanded its bed capacity, particularly in the Intensive Care Unit (ICU), restructured its physical space, reorganized patient flow, mobilized human, material, and equipment resources, and adopted other solutions to improve patient care. These efforts yielded results similar to those found in the present study^(11)^.

Regarding “Planning for Pandemic Response”, some authors highlight the role of the hospital manager as a key element in planning actions to ensure adherence to best practices. Many hospitals have adopted planning models that emphasize strategies to meet community healthcare needs, with actions based on programming, organization, direction, and control. This aligns with the findings of this study, in which managers confirmed that all planning was conducted in response to increased demand for patient care and, consequently, the expansion of necessary resources^(12)^.

An essential aspect of the planning process was the institution’s use of protocols from the H1N1 pandemic as a reference. Similarly, in South Korea, authors report that after experiencing an epidemic caused by Middle East Respiratory Syndrome (MERS), there was a restructuring of infectious disease response strategies. This prior experience facilitated adherence to preventive measures by both the population and healthcare professionals^(13)^.

Thus, the findings of this study demonstrate that senior management strategies were initiated in response to the spread of information about a global disease, of which health authorities had little knowledge—an emerging virus with an unknown etiology that was causing illness and death. This scenario aligns with the study by Barbosa et al.^(14)^, which also described the stressors faced by senior management, such as uncertainty, fear of the unknown, and concerns about contamination associated with providing care to affected populations.

At the hospital institution under study, it was necessary to implement measures to expand total bed capacity, resulting in a 29% increase compared to the capacity available before the pandemic. The literature indicates that hospitals, in general, focused primarily on expanding the number of beds and reducing regional disparities. However, paradoxically, this led to an increase in social inequalities, as most of the expansion was allocated to private beds and health insurance plans, particularly for ICU or semi-intensive care beds^(15)^. This characteristic differs from the findings of this study, as the expansion of the hospital under study was 100% dedicated to meeting the demands of the SUS, contributing to the reduction of social inequalities.

Thus, it is emphasized that, in addition to the availability of beds, another major challenge in hospital management for the specific treatment of COVID-19 was related to bed management mechanisms^(16)^.

Regarding the reorganization of human, administrative, and support resources, the concern of senior management in ensuring a sufficient number of healthcare, administrative, and support professionals was evident. In other contexts, this concern was highlighted by the shortage of human resources to combat the pandemic due to the significant increase in cases, a situation that resulted in an overwhelming demand on healthcare systems, which, even before COVID-19, were already historically overburdened, further exacerbating the situation^(17)^.

In this study, the concern of managers for the health of employees was evident, which is why medical care was expanded for HU-UEL professionals and their families at DASC.

A study analyzing the main complaints of employees attended by the psychological services provided by the employing institution found that one of the most frequently reported concerns was workers’ efforts to support their families—both in terms of financial assistance, due to the unemployment of other family members, and access to medical consultations, as these relatives had sometimes also contracted COVID-19^(18)^. In this way, the institution under study contributed not only to the employability of family members but also to specialized medical care for them.

Within the process dimension, managers reported the creation of a working group, the implementation of cohort isolation, and the use of rounds as a strategy for multiprofessional communication. It was observed that this experience was also mentioned in another hospital in the southern region of the country, where managers sought to bring together professionals, including nurses, to plan actions, define patient care and training workflows, and develop Standard Operating Procedures (SOPs), thereby improving the quality of care provided^(19)^.

The reorganization of patient care workflows for individuals affected by COVID-19 was described in a study conducted in South Korea, where a safe triage system, known as drive-through (DT), was implemented. This model was characterized as “short and fast”, comprising the following steps: entry, registration, examination, sample collection, instructions, and exit. The objective was to identify as many asymptomatic individuals as possible, as they could represent the primary source of transmission and potentially contribute to the spread of the virus in the country^(20)^.

The cohort isolation practice adopted at the institution under study consists of accommodating patients with suspected or confirmed COVID-19 in shared spaces. This approach was recommended by the Ministry of Health in cases where individual room hospitalization was not possible. When properly structured, this model could ensure safety and effectiveness in precautionary isolation. This practice was also implemented in other public hospitals^(21)^.

Regarding the multiprofessional round, also known as “multi- or interprofessional visits”, it is a tool for discussing cases of patients affected by COVID-19, allowing professionals to share decisions to promote patient safety and individualized therapeutic goals^(22)^. This strategy was adopted for the reorganization of workflows and work processes at HU-UEL during the pandemic and was identified by hospital managers as a strength, even though it was carried out informally.

Conversely, another hospital that had already implemented multiprofessional rounds before the pandemic found that maintaining this practice in a daily, in-person format was not suitable at that time. For this reason, digital devices were incorporated, allowing multiprofessional meetings to be conducted remotely^(23)^.

It is noteworthy that the collection of clinical data can facilitate the understanding of prognoses in hospitalization cases, clinical outcomes, population groups with a higher prevalence of the disease or coinfection, and hospital interventions performed, enabling more effective planning of actions across different countries^(24)^.

Within the results dimension, the reports highlighted managers’ concerns regarding societal behavior, which influenced the spread of the virus; the professional vulnerability caused by the emotional impact of deaths; the knowledge gained through experience in patient care; and the respondents’ satisfaction in knowing that they did their best to address the pandemic and that they could replicate the managerial and care reorganization measures.

Managers also expressed concern about public establishments that remained open during the pandemic. The lockdown, which represented a local measure restricting people’s movement except in cases of necessity, such as obtaining food and healthcare items, had the potential to reduce disease transmission^(25, 26)^. Thus, the managers in this study associated crowd gatherings with concerns about an even greater increase in demand for the care of COVID-19 patients.

The emotional impact of deaths was also observed in the study by Almeida et al.^(27)^, which reported that nursing professionals sometimes cried in the hallways. In this context, a study analyzing the challenges faced by nurses in dealing with the process of death and dying during the pandemic identified a gap in understanding this phenomenon, possibly due to an educational model strongly based on a biomedical approach. This gap results in psychological distress for professionals when confronting terminality, which is why the authors suggest the implementation of continuous and permanent education programs to adequately prepare these professionals^(28)^.

The managers interviewed expressed satisfaction with the reorganization measures adopted. Although there is a scarcity of literature on managers’satisfaction regarding their own management in the reorganization of managerial and care services during the COVID-19 pandemic, an integrative review of Brazilian literature that aimed to describe disease response strategies highlighted that, in general, the pursuit of solutions facilitated decision-making. Therefore, the adoption of effective and appropriate response processes can be a key element for professional satisfaction^(29)^.

### Study limitations

One limitation of this study is that data collection took place when the pandemic was already in decline, meaning that the participating managers had already gained extensive experience in managing the health crisis and had possibly minimized the challenges they had faced.

### Contributions to Public Health

The experiences of the participants in this study may contribute to the formulation of strategies for responding to future pandemics and public health challenges, providing a foundation for decision-making by managers at a global level.

## FINAL CONSIDERATIONS

Hospital reorganization in managerial and care aspects was structured based on Donabedian’s triad, whose dimensions are interrelated and act simultaneously in addressing the COVID-19 pandemic. The managers’ statements highlighted that the implemented measures not only ensured an effective workflow but also enabled the studied institution to provide high-quality care in combating the pandemic. This resulted in managers’ satisfaction due to the effectiveness of the solutions and decision-making processes.
