# Supplementary material for: Hospital reorganization at a public university hospital to address the COVID-19 pandemic
Source: Rev Bras Enferm. 2025 Sep 8;78(3):e20240281. doi: 10.1590/0034-7167-2024-0281 (PMC12419776; doi:10.1590/0034-7167-2024-0281)
Supplement: Translation

# Translation: Reorganização hospitalar de um hospital universitário público para o enfrentamento da pandemia da COVID-19

**DOI:** 10.1590/0034-7167-2024-0281pt

**Authors:** Cleonice Roseli Ribeiro, Maria do Carmo Fernandez Lourenço Haddad, Flavia Mendonça da Silva Oussaki, Natacha Bolorino, Cremilde Aparecida Trindade Radovanovic, Maynara Fernanda Carvalho Barreto, Patrícia Aroni Dadalt

**Affiliations:** I Universidade Estadual de Londrina. Londrina, Paraná, Brasil; II Universidade Estadual de Maringá. Maringá, Paraná, Brasil; I Universidade Estadual do Norte do Paraná. Bandeirantes, Paraná, Brasil

**Keywords:** Reestruturação Hospitalar, Administração Hospitalar, Hospitais Universitários, Pandemias, COVID-19

## Abstract

**Objetivos:**

evidenciar as estratégias adotadas por gestores hospitalares na reorganização dos processos gerenciais e assistenciais de um hospital universitário público para o enfrentamento da pandemia da COVID-19.

**Métodos:**

estudo de caso único, transversal e descritivo, com abordagem qualitativa. A coleta de dados foi realizada por meio de análise documental e entrevistas com gestores, conduzidas entre março e junho de 2022, com duração média de 30 minutos. Os dados foram analisados à luz dos pressupostos da Tríade de Donabedian.

**Resultados:**

participaram do estudo nove gestoras, com idade entre 40 e 60 anos e mais de cinco anos de experiência no cargo. Os eixos temáticos foram agrupados nas dimensões: estrutura, processo e resultado.

**Considerações Finais:**

o processo de trabalho foi redefinido conforme o contexto pandêmico vivenciado, evidenciando a aplicabilidade da Tríade de Donabedian ao longo de todo o processo de reestruturação assistencial e organizacional da instituição estudada.

## INTRODUÇÃO

Considerada um dos maiores desafios do século XXI, a pandemia de COVΊD-19 gerou elevada demanda de atendimento emergencial no Sistema Único de Saúde (SUS), instaurando uma crise sanitária brasileira sem precedentes. A doença disseminou-se rapidamente e acometeu pessoas em mais de 100 países dos cinco continentes, fazendo com que a Organização Mundial da Saúde (OMS) declarasse uma pandemia em 11 de março de 2020^(1)^.

O enfrentamento da pandemia da COVID-19 exigiu rápida reorganização dos serviços hospitalares, com a finalidade de assegurar um bom atendimento assistencial e gerencial e servir de retaguarda aos casos graves da doença, considerando o aumento de leitos dentro das estruturas hospitalares já existentes, bem como a ampliação e estruturação de novos hospitais de retaguarda, a fim de suprir a demanda já existente de superlotação dos hospitais^(2)^.

Nessa vertente, o Centro de Operações de Emergências em Saúde Pública (COESP) de Londrina – PR, Brasil, designou o Hospital Universitário da Universidade Estadual de Londrina (HU-UEL) para atuar como “hospital de referência” em março de 2020, para o atendimento exclusivo de casos das classificações “moderado” e “grave”, referenciados pelo Serviço de Atendimento Móvel de Emergência (SAMU), pelo Serviço Integrado de Atendimento ao Trauma e Emergência (SIATE) e pela Central Estadual de Regulação de Leitos^(3)^.

Devido ao contexto vivenciado para o enfrentamento da pandemia, os gestores e a equipe de saúde passaram por muitos desafios, o que exigiu o planejamento de políticas e práticas gerenciais eficazes para a provisão de condições adequadas ao cuidado dos usuários acometidos pela doença nos cenários hospitalares^(4)^. Com base nisso, foi constatada uma lacuna na literatura sobre estudos que descrevem a vivência dos gestores, de modo a subsidiar a gestão de futuras pandemias e emergências de saúde pública.

Diante do exposto, foi elaborada a seguinte pergunta: Como os gestores de um hospital universitário público implementaram e vivenciaram os processos de reorganização dos serviços gerenciais e assistenciais para o enfrentamento da pandemia de COVID-19?

## OBJETIVOS

Evidenciar as estratégias de gestores hospitalares na reorganização dos processos gerenciais e assistenciais em um hospital universitário público para o enfrentamento da pandemia da COVID-19.

## MÉTODOS

### Aspectos éticos

O estudo seguiu a Resolução nº 510/2016, que trata de orientações para pesquisas envolvendo seres humanos^(5)^, e foi aprovado pelo Comitê de Ética em Pesquisa com Seres Humanos. Os participantes assinaram o Termo de Consentimento Livre e Esclarecido (TCLE), e os pesquisadores assinaram o Termo de Confidencialidade e Sigilo (TCS).

### Referencial teórico-metodológico

Adotou-se, como referencial teórico e metodológico, a tríade de Avedis Donabedian, que considera as três dimensões da qualidade na área da saúde: 1) Estrutura; 2) Processos; 3) Resultados^(6, 7)^.

O primeiro componente, “estrutura”, abrange os recursos humanos, físicos e financeiros utilizados na provisão de cuidados de saúde, bem como os arranjos organizacionais e os mecanismos de financiamento desses recursos. O componente “processo” refere-se às atividades que constituem a atenção à saúde e envolvem a interação entre profissionais de saúde e a população assistida. Já os “resultados” dizem respeito às mudanças no estado de saúde da população promovidas pelos cuidados recebidos^(6, 7)^.

### Tipo de estudo

Trata-se de um estudo de caso único, desenvolvido com abordagem qualitativa e análise documental, conduzido conforme as diretrizes do *Consolidated Criteria for Reporting Qualitative Research* (COREQ)^(8)^.

### Cenário do estudo

O local do estudo foi o HU-UEL, considerado o maior órgão suplementar da Universidade Estadual de Londrina. Implantado em agosto de 1971, o HU-UEL atende pacientes de aproximadamente 250 municípios do Paraná e de mais de 100 cidades de outros estados e possui 419 leitos, totalmente à disposição do SUS, distribuídos entre diversas especialidades médicas^(3)^.

Para a realização das entrevistas, foram utilizadas as salas de cada gestor, com duração média de 30 minutos. Para preservar as informações, a porta se manteve fechada, e a entrevista contou apenas com a presença do participante e da pesquisadora, devidamente capacitada para essa finalidade.

### Fonte de dados

A população do estudo foi constituída por gestores hospitalares do HU-UEL. A amostra foi obtida por meio da técnica de amostragem “bola de neve” (*Snowball Technique*)^(9)^. Desse modo, à medida que um gestor concluía sua entrevista, era solicitado que indicasse outro gestor que havia participado do processo de reorganização da instituição para torná-la hospital de referência no atendimento à COVID-19.

### Coleta e organização dos dados

Para a realização das entrevistas, utilizou-se um formulário de caracterização demográfica e profissional, contendo informações como diretoria de atuação, sexo, faixa etária, tipo de vínculo profissional, tempo de experiência no exercício profissional e tempo de experiência na gestão hospitalar. Além disso, aplicou-se um roteiro de entrevista semiestruturada, contendo uma pergunta norteadora: “Conte-me sobre a reorganização dos processos de trabalho para o enfrentamento da pandemia da COVID-19 neste hospital.” Perguntas auxiliares foram formuladas com a finalidade de obter maior aprofundamento sobre o processo de trabalho, caso o participante não revelasse muitas informações referentes à pergunta inicial. As falas foram gravadas em um gravador de celular e em um gravador portátil.

Foi realizada a transcrição integral das entrevistas por meio do conversor de áudio em texto do *Microsoft Word*® versão 365, para a produção da primeira versão da transcrição verbal, com a confirmação da audiodescrição por dois pesquisadores independentes.

Para garantir o anonimato dos participantes, empregou-se a codificação da letra “P”, para participante, seguida de um número arábico sequencial conforme a ordem das entrevistas realizadas: PI, P2, P3 etc.

Já a coleta de dados documentais ocorreu no mesmo período, por meio do acesso à intranet denominada Rede HIT. Buscaram-se documentos gerenciais que apoiassem a compreensão do plano de contingenciamento e dos fluxos gerenciais e assistenciais internos e externos. Os documentos de interesse foram salvos em formato PDF no computador institucional e, posteriormente, em um pen drive.

### Análise dos resultados e estatística

Os dados demográficos e profissionais foram analisados com base na estatística descritiva (frequência simples), por meio do *Microsoft Excel*®. As entrevistas foram analisadas de acordo com os pressupostos de Bardin^(10)^, estruturadas em três etapas: pré-análise, exploração do material e tratamento dos resultados, emergindo três elementos de acordo com os pressupostos de Donabedian.

## RESULTADOS

Participaram deste estudo nove gestores, os quais atuavam nas diretorias: Superintendência (n = 01), Enfermagem (n = 04), Administrativa (n = 02) e Clínica (n = 02), todas mulheres, na faixa etária de 40 a 60 anos, com vínculo efetivo como servidoras públicas, experiência profissional acima de 10 anos e mais de cinco anos na função de gestora hospitalar.

A análise dos discursos revelou suas vivências no enfrentamento da pandemia de COVID-19, os quais se enquadraram em eixos temáticos e foram classificados segundo as dimensões estrutura, processo e resultado da Tríade de Donabedian^(6)^.

À luz dos pressupostos de Donabedian, evidenciados na dimensão estrutura, as entrevistadas trouxeram cinco eixos temáticos, conforme ilustram as falas a seguir.

### Momento em que o HU-UEL tornou-se referência para o atendimento de pacientes com COVID-19

Os gestores resgataram o momento histórico em que foram designados como hospital referência para o atendimento de pacientes com COVID-19, após as primeiras notícias advindas de Wuhan, na China:

*No final de 2019 fomos designados pela Secretaria Estadual de Saúde para sermos referência para o atendimento da COVID-19 no norte do Estado e dentro da rede de atenção do estado, juntos com os quatro HUs do Paraná.* (P1)

*A partir do momento em que o HU-UEL foi designado como hospital referencia para o atendimento da COVID-19, tivemos que fazer rapidamente muitas adequações nos processos de trabalho.* (P4)

### Planejamento para o enfrentamento da pandemia

Neste eixo, os participantes relataram a adaptação de protocolos adotados em pandemias anteriores e a revisão do processo de trabalho vigente.

*A chefia médica fez uma adaptação no protocolo de atendimento para pandemia H1N1 e determinou que os pacientes fossem atendidos seguindo esse protocolo.* (P5)

*O planejamento das ações, foi realizado pela revisão do processo de trabalho, desenho de novos fluxos de atendimento, reestruturação de serviços, muito treinamento, muito engajamento, responsabilidade, perseverança e confiança da equipe, para que pudéssemos de forma unida, solidificar este processo dentro do HU-UEL.* (P1)

### Reorganização da estrutura física

No início da pandemia, mesmo a estrutura física do HU-UEL sendo considerada adequada para um hospital de ensino público, terciário, de grande porte e referência para Londrina e toda a macrorregião norte do Paraná, foi necessário fazer vários ajustes em sua infraestrutura para tornar-se referência no atendimento do paciente com COVID-19.

*Assim que o hospital foi designado como hospital de referência, foi disponibilizado o financiamento, houve a ampliação do Pronto-socorro de 2.500 m2 para mais de 5.000 m2 e houve a finalização da obra da nova maternidade que serviu como hospital de retaguarda durante este período.* (P1)

Sendo assim, a estrutura física do HU-UEL, que outrora consistia em edificações de aproximadamente 40.600 m^2^, expandiu-se para aproximadamente 54.205,01 em 2020, distribuídos em um terreno com cerca de 100.000 m|2. Em 2019, a capacidade instalada da instituição para internações em diversas especialidades médicas compreendia 294 leitos, mas, ao longo da pandemia, até 2023, passou a 419 leitos (aumento de 29%), todos à disposição do SUS e distribuídos em unidades assistenciais.

No pronto-socorro ampliado, havia uma área de emergência com leitos destinados à internação ambulatorial de pacientes com acometimentos cardiovasculares. Em razão do aumento na admissão de pacientes positivados para COVID-19, foi necessária a utilização desses leitos.

Já o anexo hospitalar designado para a nova maternidade, prevista para ser inaugurada em 2020, foi montado como hospital de retaguarda, com capacidade para 100 leitos, devido à necessidade de ampliação para o internamento de pacientes com COVID-19.

### Reorganização dos recursos humanos, administrativos e de apoio

Para atender ao aumento da demanda de serviços, foi necessário contratar vários profissionais, tanto da área da saúde quanto de outras áreas correlatas (apoio), na modalidade de prestadores de serviços, via chamamento público, e realocar outros servidores.

Antes da pandemia, a instituição contava com 355 servidores públicos estatutários e 261 prestadores de serviços, totalizando 616 colaboradores. Para atender às novas necessidades, esse número aumentou para 472 servidores públicos e 777 terceirizados, totalizando 1.249 trabalhadores, o que representou um aumento de 101% no contingente de recursos humanos em atuação.

Durante a pandemia, os holofotes se voltaram principalmente para os profissionais da saúde, mas muitos trabalhadores administrativos e de apoio estavam presentes no ambiente hospitalar e foram fundamentais para o êxito do cuidado em saúde, como destaca a fala de uma participante:

*O pessoal do transporte administrativo que nunca havia colocado a mão ou transportado um paciente em óbito, vieram ajudar, sendo que são motoristas administrativos e eles tiveram que lidar com isso.* (P7)

Observa-se que tanto o aumento do quantitativo dos recursos humanos quanto a ampliação do escopo de atribuições laborais dos profissionais administrativos foram medidas de reorganização estrutural.

### Implantação de serviços novos

Precisou-se ampliar a Divisão de Assistência à Saúde da Comunidade (DASC), serviço que foi implementado nas dependências do hospital em 2002, para prestar serviços de atenção à saúde da comunidade universitária e dos colaboradores, oferecendo assistência de clínica médica, enfermagem e odontologia, por meio de atendimentos eletivos ou de baixa complexidade. Essa preocupação com os trabalhadores fica clara nos seguintes relatos:

*Uma instituição não tem como se desenvolver se não cuida dos seus profissionais e então a DASC recebeu investimento, bem como foram contratados um psiquiatra e um cardiologista para o atendimento dos colaboradores durante a pandemia.* (P5)

*A DASC que nos atendeu, até os nossos parentes, então nós tínhamos aquele privilégio, de não precisar ficar lá naquelas filas longas em busca de atendimento.* (P5)

Na dimensão processo, foram identificados dois eixos temáticos, descritos a seguir.

### Reorganização dos fluxos e dos processos de trabalho

Diversos fluxos foram implantados, alterando o processo de trabalho, desde a entrada no pronto-socorro para os casos de COVID-19 até o atendimento e o encaminhamento para os setores internos. Essas mudanças foram resultado do trabalho de um grupo de profissionais especialistas designado para essa finalidade, além da implementação do isolamento em coorte dos indivíduos com diagnóstico positivo e negativo. Outra estratégia mencionada nas entrevistas foi a adoção de um processo de comunicação multiprofissional deliberativo (*round*), voltado para a discussão de boas práticas gerenciais e assistenciais.

*Foi montado um grupo de trabalho, de diversas áreas, maioria chefias, mas também muitos especialistas e era ali que decisões eram tomadas, muitas reuniões administrativas para definir novos fluxos de atendimento e de realização de exames, reunião de como que seria os critérios para a porta de entrada, a capacitação dos recursos humanos porque nós não sabíamos também como iria ficar.* (P4)

*Tentamos organizar um fluxo, porque estávamos desorganizados, fomos separando os locais para organizar onde o paciente chegaria, quem era considerado suspeito, quem era considerado baixo suspeita de acordo na época da tomografia do paciente, bem como separar os pacientes positivos, negativos, separar alta suspeita fizemos um fluxo bem organizado.* (P9)

*Essa reorganização foi feita através de revisão de processo, de desenho de fluxo de reestruturação de serviços, muita capacitação, muito engajamento, responsabilidade, perseverança e confiança na equipe, para que nós pudéssemos de forma unida, solidificar este processo dentro do HU-UEL.* (P1)

*O trabalho em conjunto, foi o que nos salvou, teve uma interação maior entre as equipes, o “round” ajudou muito a comunicação entre os membros da equipe. Eu acho que isso foi fundamental para que conseguíssemos realizar os atendimentos.* (P9)

### Coleta de informações sobre as características clínicas da COVID-19

Os pacientes acometidos pela COVID-19 apresentaram características clínicas com diferentes níveis de gravidade, de acordo com a faixa etária, nos momentos relatados pelos participantes. Além disso, foi revelado que houve alternância na evolução para quadros graves, variando conforme a faixa etária, bem como o surgimento de condições clínicas inesperadas pela equipe assistencial.

*Houve momentos que tivemos a infecção pela COVID-19 que foi totalmente diferente do que aconteceu, foi um quadro grave para os pacientes idosos e gravíssimos para os pacientes jovens, o que foi muito difícil para todo mundo ver pacientes jovens serem intubados e muitos deles morrerem, isso foi um de problemas e os residentes choravam muito.* (P9)

[...] *os pacientes com a COVID-19, tinham uma hipoxemia considerada silenciosa e então o paciente estava com uma queda na saturação e acordado e às vezes não tinha a percepção que estava com falta de ar ou não tínhamos a percepção da gravidade dele e da dispneia.* (P9)

Na dimensão resultados, emergiu somente um eixo temático.

### Dificuldades, sentimentos e aprendizagens vivenciados no processo de gestão da pandemia de COVID-19

Foram mencionadas fragilidades relacionadas aos processos de trabalho da alta gestão e de manutenção do trabalho em equipe, pois um dos gestores informou ainda estar no início do seu mandato quando precisou enfrentar esse desafio.

*Foi uma gestão muito conturbada em todos os sentidos desde o começo tivemos várias intercorrências administrativas e aí veio a pandemia, e então já fomos desafiados a enfrentar um problema grave.* (P2)

Também foram realçados os sentimentos de outros gestores, de profissionais da saúde e de membros da sociedade.

*Academias e bares em funcionamento e as pessoas sendo internadas e graves, eu comecei a chorar. Meu Deus, eu não vou dar conta.* [...] *Eu não tenho mais onde colocar os corpos e os respiradores estão acabando e as pessoas estão no bar, bateu desespero, eu entrei em pânico.* (P7)

*Os funcionários criaram Choródromo* [ponto de encontro para chorar] *no Pronto Socorro, porque era um lugar que os funcionários iam chorar.* (P7)

*No momento de diminuição dos novos casos de COVID-19, além do legado da ampliação da infraestrutura e aquisição de equipamentos, ficou todo o aprendizado vivenciado nesse período.* (P9)

*Se surgir outra pandemia, nós vamos vencer, vamos fazer tudo igual.* (P2)

Diante dos relatos, ficou evidente que o enfrentamento da COVID-19 somente foi possível devido ao estabelecimento de estratégias emergentes, que geraram aprendizados e superação das dificuldades vivenciadas.

## DISCUSSÃO

A análise do processo de reorganização dos serviços gerenciais e assistenciais na instituição em estudo foi permeada por ações da alta gestão e pelo planejamento de novos fluxos do processo de trabalho.

Donabedian^(6)^ considera a dimensão estrutura a mais importante, pois esta pode influenciar as demais, reforçando a relevância de seu monitoramento, incluindo todos os aspectos organizacionais e os recursos materiais.

Destaca-se que, durante o período pandêmico, a instituição estudada adequou e reorganizou suas estruturas para atender pacientes com a síndrome respiratória, além de repensar o modelo convencional de cuidado presencial. Isso resultou na implementação de várias estratégias assistenciais, com o objetivo de otimizar o atendimento e reduzir a propagação da contaminação, sem prejuízo dos tratamentos necessários.

Nessa vertente, o Hospital das Clínicas da Faculdade de Medicina de Ribeirão Preto (HCFMRP-USP) também foi nomeado um dos hospitais de referência para o tratamento de casos de COVID-19, exigindo uma reestruturação interna para atender à demanda da região. Para isso, o hospital ampliou leitos, especialmente os da Unidade de Terapia Intensiva (UTI), readequou sua estrutura física, reorganizou os fluxos de pacientes, mobilizou recursos humanos, materiais e equipamentos e adotou outras soluções para a prestação de cuidados aos pacientes, obtendo resultados similares aos do presente estudo^(11)^.

Quanto ao “Planejamento para o enfrentamento da pandemia”, alguns autores destacam o papel do gestor hospitalar, que passa a ser visto como elemento fundamental para o planejamento das ações que garantirão o cumprimento das melhores práticas. Muitos hospitais têm adotado um modelo de planejamento com ênfase nas estratégias de atendimento às necessidades da comunidade, sobretudo na área da saúde, com ações baseadas em programação, organização, direção e controle. Isso corrobora os achados deste estudo, no qual os gestores reafirmaram que todo o planejamento foi conduzido de acordo com o aumento da demanda nos atendimentos e, consequentemente, com o crescimento das necessidades^(12)^.

Um ponto essencial do planejamento foi o uso, pela instituição, dos protocolos empregados na pandemia do H1N1 como norteadores. De forma similar, no Sul da Coreia, autores relatam que, após a experiência com a epidemia causada pela Síndrome Respiratória do Oriente Médio (MERS), houve uma reestruturação no enfrentamento de doenças infecciosas, e essa vivência prévia favoreceu a adesão às medidas por parte da população e dos profissionais^(13)^.

Portanto, os achados deste estudo demonstram que as estratégias de atuação da alta gestão foram iniciadas diante da propagação de informações sobre o contexto mundial de uma doença sobre a qual as autoridades sanitárias tinham pouco conhecimento — um vírus de etiologia ainda desconhecida, que estava adoecendo e levando pessoas a óbito. Esse cenário vai ao encontro do estudo de Barbosa et al.^(14)^, que também descreveu os agentes estressores enfrentados pela alta gestão, tais como insegurança, medo do desconhecido e preocupação com a contaminação associada ao atendimento da população acometida.

Na instituição hospitalar estudada, foi necessário adotar medidas para ampliar a capacidade total de leitos, o que resultou em um aumento de 29% em relação à capacidade ofertada antes da pandemia. A literatura aponta que os hospitais, de modo geral, limitaram-se à expansão no número de leitos e à redução das desigualdades regionais, o que, paradoxalmente, gerou um aumento das desigualdades sociais, pois essa ampliação, em sua maioria, foi destinada a leitos particulares e a planos de saúde, especialmente os de UTI ou de cuidados semi-intensivos^(15)^. Essa característica difere dos resultados encontrados nesta pesquisa, uma vez que a ampliação do hospital estudado foi 100% destinada ao atendimento das demandas do SUS, contribuindo para a redução das desigualdades sociais.

Sendo assim, reforça-se que, além da disponibilização de leitos, outro grande desafio da gestão hospitalar para o tratamento específico da COVID-19 esteve associado ao mecanismo de gestão de leitos^(16)^.

Acerca da “Reorganização dos recursos humanos, administrativos e de apoio”, ficou clara a preocupação da alta gestão em disponibilizar profissionais da área da saúde, administrativos e de apoio em número suficiente. Em outras realidades, essa preocupação foi evidenciada pela escassez de recursos humanos para o enfrentamento da pandemia diante do aumento significativo de casos, fato que resultou em uma demanda devastadora para os sistemas de saúde, os quais, antes mesmo da COVID-19, já estavam historicamente sobrecarregados, situação que se agravou ainda mais^(17)^.

Neste estudo, ficou evidente a preocupação dos gestores com a saúde dos colaboradores, motivo pelo qual foram ampliados os atendimentos médicos para os profissionais do HU-UEL e para seus familiares na DASC.

Em um estudo que analisou as principais queixas dos colaboradores atendidos pelo serviço de psicologia ofertado pela instituição empregadora, um dos discursos mais frequentes foi a preocupação dos trabalhadores em auxiliar suas famílias, tanto no suporte à renda familiar, devido ao desemprego de outros membros, quanto na disponibilização de consultas médicas, visto que, por vezes, esses familiares também haviam contraído a COVID-19^(18)^. Dessa maneira, considera-se que a instituição analisada contribuiu tanto para a empregabilidade dos familiares quanto para o atendimento médico especializado da família.

Na dimensão processo, os gestores relataram a criação de um grupo de trabalho, a implementação do isolamento em coorte e a realização de *rounds* como estratégia de comunicação multiprofissional. Observou-se que essa experiência também foi mencionada em outro hospital da região Sul do país, onde os gestores buscaram reunir os profissionais, incluindo enfermeiros, para planejar ações, definir fluxos de atendimento e treinamento e elaborar Protocolos Operacionais Padrão (POP), qualificando assim a assistência prestada^(19)^.

A reorganização dos fluxos de atendimento de pacientes acometidos pela COVID-19 foi descrita em um estudo realizado na Coreia do Sul, no qual se implementou um sistema de triagem seguro, denominado *drive-through* (DT), caracterizado como um modelo “curto e rápido”, contendo as seguintes etapas: entrada, registro, exame, coleta de amostras, instruções e saída. O objetivo era identificar o maior número possível de indivíduos assintomáticos, que poderiam representar a principal fonte de transmissão e, possivelmente, estar colaborando para a disseminação do vírus no país^(20)^.

O isolamento em coorte, prática adotada na instituição estudada, consiste em acomodar pacientes com suspeita ou confirmação de COVID-19 em espaços comuns, sendo recomendado pelo Ministério da Saúde nos casos em que a internação em quartos individuais não fosse possível. Afinal, quando bem estruturado, esse modelo poderia garantir segurança e efetividade no isolamento de precaução. Esse padrão também foi adotado em outros hospitais públicos^(21)^.

No que tange ao *round* multiprofissional, também conhecido como “visitas multi ou interprofissionais”, trata-se de uma ferramenta para discussão de casos de pacientes acometidos pela COVID-19, permitindo o compartilhamento de decisões profissionais em prol da segurança do paciente e de um objetivo terapêutico individualizado^(22)^. Essa estratégia foi adotada para a reorganização dos fluxos e dos processos de trabalho no HU-UEL durante a pandemia, sendo apontada pelos gestores hospitalares como uma fortaleza, ainda que ocorresse de maneira informal.

Em contrapartida, outra instituição hospitalar, que já adotava os *rounds* multiprofissionais antes da pandemia, verificou que a manutenção dessa prática de forma presencial e diária não era adequada para aquele momento. Por isso, incorporaram-se dispositivos digitais, possibilitando que as reuniões multiprofissionais ocorressem remotamente^(23)^.

Salienta-se que a obtenção de dados clínicos pode favorecer a compreensão dos prognósticos nos casos de hospitalização, dos desfechos clínicos, dos grupos populacionais com maior prevalência da doença ou com coinfecção, além das intervenções hospitalares realizadas, permitindo um planejamento mais eficaz das ações em diferentes países^(24)^.

Na dimensão resultados, os relatos evidenciaram a preocupação dos gestores com o comportamento da sociedade, que interferiu na disseminação do vírus; a vulnerabilidade profissional causada pela sensibilização diante dos óbitos; o aprendizado adquirido com a experiência no atendimento; e a satisfação dos depoentes ao terem a certeza de que fizeram o melhor para o enfrentamento da pandemia e de que poderiam replicar as medidas de reorganização gerencial e assistencial.

Os gestores ainda expressaram preocupação com os estabelecimentos públicos que permaneceram abertos durante a pandemia. O *lockdown,* que representou uma medida local de restrição da circulação de pessoas, exceto em casos de necessidade, como a busca por itens de alimentação e saúde, teve o potencial de reduzir a transmissão da doença^(25, 26)^. Dessa forma, os gestores deste estudo associaram a aglomeração de pessoas à preocupação de um aumento ainda maior da demanda por atendimento de pacientes acometidos pela COVID-19.

A sensibilização diante dos óbitos também foi observada no estudo de Almeida et al.^(27)^, no qual se relatou que profissionais de enfermagem, em alguns momentos, choravam pelos corredores. Nesse contexto, um estudo que analisou os desafios do enfermeiro ao lidar com o processo de morte e morrer durante a pandemia verificou uma lacuna de compreensão desse fenômeno, possivelmente proveniente de um modelo de formação fortemente pautado na abordagem biomédica. Essa lacuna gera sofrimento psíquico nos profissionais ao enfrentarem a terminalidade, razão pela qual os autores sugerem a implementação de programas de educação continuada e permanente para o preparo adequado desses profissionais^(28)^.

Os gestores depoentes indicaram satisfação com as medidas de reorganização adotadas. Embora haja uma escassez na literatura sobre a satisfação dos gestores com relação ao próprio gerenciamento na reorganização gerencial e assistencial durante o enfrentamento da pandemia de COVID-19, uma revisão integrativa da literatura brasileira que visava descrever estratégias de enfrentamento da doença evidenciou que, de modo geral, a busca por soluções favoreceu a tomada de decisões, logo a adoção de processos de enfrentamento efetivos e adequados pode ser um elemento-chave para a satisfação profissional^(29)^.

### Limitações do estudo

Como limitação do estudo, destaca-se que a coleta de dados ocorreu quando a pandemia já estava em declínio, ou seja, quando os gestores participantes já haviam adquirido ampla experiência na gestão da crise sanitária e, possivelmente, minimizado os desafios enfrentados.

### Contribuições para a Saúde Pública

As vivências dos participantes deste estudo podem contribuir para a formulação de estratégias de enfrentamento em futuras pandemias e em desafios de saúde pública, subsidiando gestores em nível global na tomada de decisões.

## CONSIDERAÇÕES FINAIS

A reorganização hospitalar no âmbito gerencial e assistencial foi estruturada com base na tríade de Donabedian, cujas dimensões são inter-relacionadas e atuam simultaneamente no enfrentamento da pandemia da COVID-19. Os depoimentos dos gestores evidenciaram que as medidas implementadas, além de garantir um processo de trabalho eficaz, possibilitaram que a instituição estudada prestasse uma assistência qualificada no combate à pandemia, resultando em satisfação por parte dos gestores devido à resolutividade e à tomada de decisões eficazes.
